# CIN2+ detection of the HPV DNA Array genotyping assay in comparison with the Cobas 4800 HPV test and cytology

**DOI:** 10.1186/s12985-019-1197-6

**Published:** 2019-07-23

**Authors:** Aleksandra Pesic, Amrei Krings, Matthias Hempel, Rosemarie Preyer, Kimon Chatzistamatiou, Theodoros Agorastos, Andreas M. Kaufmann

**Affiliations:** 1 Gynaecology Clinic, Charité Universitätsmedizin, Corporate member of Freie Universität Berlin, Humboldt-Universität Berlin and Berlin Institute of Health, Hindenburgdamm 30, 12200 Berlin, Germany; 2 AID/GenID Diagnostika, Ebinger Strasse 4, 72479 Strassberg, Germany; 3 1st Department of Obstetrics and Gynecology, Papageorgiou Hospital, Aristotle University of Thessaloniki, Ring road, Nea Efkarpia, 56403 Thessaloniki, Greece; 4 IVth University Clinic of Obstetrics and Gynaecology, Hippokration General Hospital, Aristotle University, 49 Konstantinoupoleos St, 54642 Thessaloniki, Greece

**Keywords:** Cervical Cancer, HPV assay, HPV detection, Human papillomavirus, Validation

## Abstract

**Background:**

HPV DNA Array is an E1-targeting PCR genotyping test, with capability of distinguishing 18 high-risk (16, 18, 26, 31, 33, 35, 39, 45, 51, 52, 53, 56, 58, 59, 66, 68, 73, 82) and 11 low-risk HPV types (6, 11, 40, 42, 44, 54, 67, 69, 70, 85, 97). HPV DNA Array uses multiplex PCR for E1-gene sequence amplification. The amplicons are detected and genotyped by reverse hybridization to immobilized DNA probes spotted as triplets in single 96 well-plate wells and read by AID ELISPOT reader.

**Methods:**

Aim of the study was to evaluate the clinical performance of the assay against internationally accepted and FDA approved Cobas 4800 HPV test (Roche Diagnostics). Study population comprised of 500 cervical samples.

**Results:**

HPV DNA Array demonstrated a very high sensitivity of 100% for CIN2+ and 100% for CIN3+ detection, same as Cobas 4800. HPV DNA Array showed greater sensitivity for CIN2+ detection than cytology (100% vs. 13.6%). The agreement to Cobas 4800 for HPV detection, irrespective of type, was 81.4% with κ = 0.613. The agreement for HPV 16 was 92.8% (κ = 0.929), and for HPV 18 54.2% (κ = 0.681).

**Conclusion:**

HPV DNA Array demonstrated good clinical performance for detection of high-grade lesions, and may be considered for usage in a screening setting.

## Background

Cervical cancer, caused by persistent HPV infection [1], is an easily preventable disease. With the introduction of mass cervical cancer screening, a significant 75% decline in cancer incidence has been observed in developed countries, achieved through regular cytological screening [2, 3]. However, cytology is a method hard to implement in developing countries and has a variable sensitivity for disease detection (44–78%) [4]. The recent advancement of detection methods, and the causal link between HPV and cervical cancer, has led to a change in paradigm. HPV testing advanced from usage as a triaging method to a method for primary cervical cancer screening, approved by WHO [5] and FDA. In 2014, the Cobas 4800 HPV test (Roche Diagnostics) was approved by FDA for primary cervical cancer screening in the USA [6]. The following year, the American Society for Colposcopy and Cervical Pathology included the HPV genotyping tests as primary tests (without cytology) for cervical cancer screening in its guidelines [7].

There is evidence that HPV-based screening is more sensitive in detecting high grade lesions [8–10], and is clinically more informative. HPV-based screening could give prognostic information e.g. positivity for high-risk (HR) HPV at 6 months after lesion treatment, can predict lesion recurrence [11]. Also, women with HPV persistence of more than 7 years have a higher risk of lesion development and progression to invasive cancer [12]. Additionally HPV genotyping could provide more clinical information as not all HPV types have the same risk for cancer development, e.g. HPV 16 more than other HR-HPV types; multiple HPV infections more than single infections [13, 14].

The main objective of this study was to report on clinical performance of HPV DNA Array in detection of cervical lesions and to compare its analytical capability to Cobas 4800 HPV test on a study population comprised of 500 cervical samples.

HPV DNA Array is a full HPV genotyping assay, newly developed by AID Diagnostika GmbH (Strassberg, Germany), which is capable of genotyping separately for 29 HPV types (16, 18, 26, 31, 33, 35, 39, 45, 51, 52, 53, 56, 58, 59, 66, 68, 73, 82, and 6, 11, 40, 42, 44, 54, 67, 69, 70, 85, 97).

## Materials and methods

### Study population and sample preparation

Five hundred consecutive taken samples of approx. 4000 cervical scrapings collected for the “HEllenic Real life Multicentric cErvical Screening” (HERMES), a study that compared cytology and HPV-based screening [15] were selected. Samples were collected from women undergoing routine cervical screening at 9 different outpatient Clinics in Greece (Athens, Thessaloniki, Larisa, Patras and Alexandropoulis). Cervical scrapings were taken with Cervex Brush (Rovers Medical Devices, Oss, Netherlands) and rinsed in ThinPrep. One part of the sample was used for cytology, one part for HPV testing with Cobas HPV test, and the leftover volume of 500 samples was stored at + 4 °C and sent to Charité-Universitätsmedizin Berlin, Germany. One sample was not included in the shipment, hence, 499 samples were analyzed.

For this study, 2 ml of each sample was used for DNA extraction by QIAamp DNA Mini Kit, in accordance with manufacturer’s instructions. Nucleic acid was eluted in a final volume of 160 μl.

### HPV DNA Array

HPV DNA Array detects multiplex PCR amplified E1-gene sequences by a reverse hybridization reaction in a 96 well microtiter plate. Oligonucleotide probes, specific for each HPV type, are spotted on a bottom of each well. After hybridization, colored spots were evaluated by ELISpot reader and reading software AiDot (AID Diagnostika GmbH, Strassberg, Germany). HPV DNA Array is capable of genotyping 18 high-risk (16, 18, 26, 31, 33, 35, 39, 45, 51, 52, 53, 56, 58, 59, 66, 68, 73, 82) and 11 low-risk HPV types (6, 11, 40, 42, 44, 54, 67, 69, 70, 85, 97).

The person performing the HPV DNA Array testing was blinded to the Cobas HPV results, cytology and histology status of the samples collected.

The assays use different target sequences. While Cobas 4800 relies on the more conserved genomic region of L1 the HPV array detects sequences of the E1 that is a larger and more diverse and has more opportunities for type-specific probe sequences and a wider inclusion of genotypes in a multiplexed PCR assay.

### Cobas 4800 HPV test

The Cobas 4800 HPV is a L1-based PCR test with capability of separately genotyping HPV 16 and − 18, and grouping other 12 HR-HPV types (31, 33, 35, 39, 45, 51, 52, 56, 58, 59, 66 and 68) in a single signal.

HPV testing was performed as described by Castle et al. [16] at the Laboratory of Microbiology, Democritus University of Thrace, Alexandroupolis, Greece. The performer was blinded to cytology and histology results.

### Cytology

The cytological smear examination was performed at the corresponding pathology laboratory of the participating hospital where sample was taken. Bethesda 2001 cytology classification guideline was followed [17]. The cytologists were blinded to the HPV DNA test results.

### Sample analysis

Agreement, sensitivity, specificity, positive predictive value (PPV) and negative predictive value (NPV) were used as main outcomes. Cohen’s Kappa values determined the agreement between the assays and were interpreted as follows [18]: poor (< 0.20), fair (0.21–0.40), moderate (0.41–0.60), good (0.61–0.80), very good (0.81–1.00). The values of McNemar’s test were used to evaluate if significant discrepancies exist.

Cobas 4800 HPV test gives information on HPV positivity by genotyping separately HPV 16 and HPV 18, and reporting the results for 12 other HR-HPV types in a pool (31, 33, 35, 39, 45, 51, 52, 56, 58, 59, 66, and 68), hence, the HPV DNA Array detected HPV types were grouped accordingly for comparative analytical purposes. Only 14 HPV types covered by both assays were included in the analysis (HPV 16, 18, 31, 33, 35, 39, 45, 51, 52, 56, 58, 59, 66, and 68).

Statistical analysis was performed with IBM SPSS Statistics for Windows (Version 21.0. IBM Corp. Armonk, NY) and MedCalc 15.8 (MedCalc Software, Ostend, Belgium).

## Results

### Characteristics of the study population

Four hundred ninety-nine samples were received from the HERMES study from women aged 19 to 66 years, with an average age of 33 years. 217 women were younger than 30 years, 276 were 30 years or older, and for 6 samples, age information was unavailable. Ninety-five samples were HPV negative by Cobas HPV test, and 404 were HPV positive. HPV 16 was detected in 97 samples, HPV 18 in 48 samples, and 321 samples were positive for 12 other HR-HPV types.

Cytology result was obtained from 360 women: 274 had normal cytology, 43 had ASCUS, 35 LGSIL and 8 had HGSIL. Biopsy was taken for 74 women: 23 had normal histology, 29 had CIN1, 17 had CIN2 and 5 had CIN3 lesions. No cases of cervical cancer were reported in this population.

### HPV DNA Array results and sample re-testing

All 499 samples were tested with HPV DNA Array. An HPV negative result was found in 146 samples, and 353 samples were HPV positive.

A discrepancy to Cobas was observed in 90 samples (HPV DNA Array+/Cobas-, HPV DNA Array−/Cobas+ or both assays positive with different HPV types). To exclude test execution mistake, the 90 samples were re-tested with HPV DNA Array, along with 10 concordant samples, as control. All control samples had the same concordant results. In 82 samples, the result stayed the same, still discordant to Cobas, and in 8 samples, the result changed. To confirm which result was true, a third testing was performed, and only HPV types found in 2 or more tests were counted as truly positive. Four samples were initially HPV negative and now became HPV positive, matching the Cobas result; three samples were LR-HPV positive and now showed a co-infection with HR-HPV, and one sample was initially HPV positive for HPV 16, but after re-testing it was twice HPV negative.

In summary, after re-evaluation, HPV DNA Array deemed 143 samples to be HPV negative and 356 samples, as HPV positive, among which 25 samples only for types not detected by Cobas, e.g. HPV 42, − 53, − 54, and − 67, hence these samples were additionally placed in the HPV negative group for analysis.

We present the analysis on the re-evaluated results.

### CIN2+ lesion detection

HPV DNA Array showed a sensitivity for detecting CIN2+ lesions of 100% (95% CI, 84.6–100%), with a specificity of 9.43% (95% CI, 3.1 to 20.7%), PPV 31.43% (95% CI, 20.8 to 43.6%), and NPV 100% (95% CI, 47.8 to 100%) (Table 1). Similarly we observed a sensitivity of 100% for CIN3+ detection with all 5 lesions detected by HPV DNA Array.

**Table 1:** HPV detection stratified to histology and cytology

		HPV DNA Array		Cobas		Cytology			
Histology	Total	HPV+	%	HPV+	%	Normal	ASCUS	LGSIL	HGSIL
Normal	23	18	78.2%	23	100%	17	3	2	1
CIN1	29	24	82.7%	29	100%	19	4	6	0
CIN2	17	17	100%	17	100%	11	0	3	3
CIN3	5	5	100%	5	100%	3	1	1	0
CIN2+	22	22	100%	22	100%				13.6%

Cobas test had a sensitivity for detection of CIN2+ lesions of 100% (95% CI, 84.6–100%), with a specificity of 0% (95% CI, 0 to 6.7%).

A difference between the assays was observed only for detection of low-grade lesions approx. 80% vs. 100% of Cobas, leading to a lower specificity of Cobas test.

When compared against cytology (Table 1), HPV DNA Array demonstrated a much better sensitivity for CIN2+ detection, 100% of HPV DNA Array vs. 13.6% of cytology. Only 4 of 22 CIN2+ lesions were classified as HGSIL. Conversely, specificity was much higher with cytology 84.9% (95% CI, 72.4 to 93.2%) than with HPV DNA Array 9.43% (95% CI, 3.1 to 20.7%).

### HPV detection

HPV DNA Array was positive in 66.3% (331/499) cases, as compared with 81% (404/499) of Cobas HPV test (Table 2).

**Table 2:** HPV detection between HPV DNA Array and Cobas, stratified by age, histology, and cytology

		Cobas					
	HPV DNA Array	Positive	Negative	Agreement	Kappa	Interpretation^a^	McNemar’s p
Overall population (499)	Positive	329	2	81.4%	0.613	good	0.000
	Negative	75	93				
< 30 (217)^b^	Positive	163	0	86.2%	0.618	good	0.000
	Negative	26	28				
≥30 (276) ^b^	Positive	164	2	77.4%	0.593	moderate	0.000
	Negative	48	62				
CIN2+ (22)	Positive	22	0	100%			
	Negative	0	0				
HGSIL (8)	Positive	8	0	100%			
	Negative	0	0				

^a^Interpretation values: poor (< 0.20), fair (0.21–0.40), moderate (0.41–0.60), good (0.61–0.80), very good (0.81–1.00)

^b^6 samples, for which the age information was unavailable, were excluded from the analysis

The agreement between the assays was 81.4% (95% CI, 80.8 to 87.5%) with κappa 0.613 (95% CI, 53.9 to 68.7%). The results were stratified according to age; sensitivity, agreement for HPV detection and specificity within < 30 group were 86.2% (95% CI, 80.5 to 90.8%), 0.618 (95% CI, 49.1 to 74.5%), 100% (95% CI, 87.6 to 100%), and within ≥30 group 77.4% (95% CI, 84.6 to 100%), 0.593 (95% CI, 49.9 to 68.8%) and 96.87% (95% CI, 89.2 to 99.6%), respectively. Values of the McNemar’s test deemed the differences statistically significant (*p* = 0.000).

Further on, when focusing on agreement among CIN2+/HGSIL lesions, an agreement of 100% was observed.

### HPV partial genotyping

Cobas test genotypes HPV 16 and − 18 separately, and groups the results of 12 HR-HPV types in a pool (31, 33, 35, 39, 45, 51, 52, 56, 58, 59, 66, and 68), hence, the HPV DNA Array results were adjusted accordingly.

HPV 16 was detected in 90 samples by HPV DNA Array, as compared with 97 found by Cobas (Table 3), leading to a sensitivity of 92.8% (95% CI, 85.7 to 97%) and kappa agreement of 0.929 (95% CI, 88.7 to 97%), with specificity 99.0% (95% CI, 97.5 to 99.7%), and PPV and NPV 95.74% (95% CI, 89.5 to 98.8%) and 98.27% (95% CI, 96.5 to 99.3%). McNemar’s test value was greater than 0.05.

**Table 3:** HPV genotype detection of HPV DNA Array in comparison with Cobas

	Cobas	HPV DNA Array	Agreement	Kappa	Interpretation^a^	McNemar’s p
HPV 16	97	90	92.8%	0.929	very good	0.549
HPV 18	48	26	54.2%	0.681	good	0.000
12 other HR-HPV^b^	321	242	75.4%	0.677	good	0.000

^a^ Interpretation values: poor (< 0.20), fair (0.21–0.40), moderate (0.41–0.60), good (0.61–0.80), very good (0.81–1.00);

^b^ HPV 31, 33, 35, 39, 45, 51, 52, 56, 58, 59, 66, and 68

HPV DNA Array detected HPV 18 in 26 samples vs. 48 found by Cobas, demonstrating a sensitivity of 54.2% (95% CI, 39.2 to 68.6%) with moderate agreement (κ = 0.681, 0.558 to 0.805), specificity of 100% (95% CI, 99.2 to 100%) and PPV and NPV of 100% (95% CI, 86.% to 100) and 95.3% (95% CI, 93 to 97%). McNeimar’s test value was less than 0.05.

Twenty-two HPV 18 Cobas positive samples were not detected by HPV DNA Array. Thirteen were single infections by Cobas, and 9 samples were co-infections with HPV 16 and/or other HR-HPV, all of which were detected by HPV DNA Array and only HPV 18 was missed in the multiple infections.

Histology was available for 6 samples of 22 missed. We observed that only one sample had a histologically confirmed high-grade lesion (CIN2) and was a Cobas detected multiple infection. In that case, HPV DNA Array failed to detect HPV 18, but other HR-HPV types present in the infection were found.

Sensitivity for detecting 12 other HR-HPV types was 75.4% (95% CI, 70.3 to 80%) with 242 samples detected as compared with 321 by Cobas. Kappa showed good agreement of 0.677 (95% CI, 61.6 to 73.9%). Specificity, PPV and NPV were 98.88% (95% CI, 96 to 99.9%), 99.18% (95% CI, 97 to 99.9%) and 69.02% (95% CI, 62.9 to 74.6%). The difference was graded statistically significant (McNemar’s *p* <  0.05).

HPV DNA Array was negative for other HR-types in 75 cases, whereas Cobas was positive. Sixty-five were single infections, and ten samples were co-infections with HPV 16. In all cases HPV DNA Array detected HPV 16. Histology was available for only 7 of the 75 samples. Six samples had a low grade lesion (<CIN2), and in one case a high-grade lesion (CIN3) was confirmed. In that case Cobas showed a co-infection of 12 other HR types with HPV 16. HPV DNA Array did detect HPV 16, missing to detect other HR-HPV types.

## Discussion

Main aim of this study was to investigate the HPV DNA Array’s ability to detect high-grade lesions by comparing its performance with Cobas HPV test, a validated and FDA approved assay for primary screening, and with cytology, an established method for cervical cancer screening.

HPV DNA Array was found to be a simple and short assay (assay execution was in total four hours, with a hands-on time of approx. two hours). It included a reverse hybridization step, and an ELISA-like staining for assay development. The readout was automated with the ELISPOT reader and an AiDot software that evaluated the full 96 well plate in approx. three minutes. HPV DNA Array is a time efficient assay that allows high throughput, which could be of great benefit for mass screening. Additionally, the HPV DNA Array could be used for epidemiology of HPV, as it fully genotypes and includes most prevalent LR-HPV types (Manuscript in preparation).

In this study, HPV DNA Array demonstrated a very high clinical performance with a sensitivity for detecting CIN2+/3+ lesions of 100%, identical to the clinical sensitivity of Cobas HPV test, and higher than cytology (13.6%) in this population. The Cobas HPV test is well investigated and its performance is well documented in the literature. Other researchers reported a similar performance [19, 20]. However, surprisingly, a low sensitivity of cytology, 13.6%, with only 3 of 22 CIN2+ lesions classified as HGSIL, was found. Although it has been documented that HPV assays are more sensitive for disease detection in comparison with cytology [21], such difference was not expected. Koliopoulos et al. [21] reported a sensitivity of cytology of approx. 60%, however, in this study, it was only 13.6%.

Looking at agreement for HPV detection, irrespective of type, HPV DNA Array showed a good agreement of 81.4% (k = 0.613) as compared with Cobas. This difference was rated statistically significant by McNemar’s test (*p* <  0.05), nonetheless, since all CIN2+ cases were detected, the difference had no clinical meaning.

Analyzing the agreement for specific HPV types, a high sensitivity for detection of HPV 16 was observed (>90%, k = 0.929), which is the most common cancer causing genotype [22]. A lower sensitivity for HPV 18 detection (54.2%, κ = 0.681, *p* <  0.05), and for detection of other 12 HR-types (75.4%, κ = 0.677, *p* <  0.05) was found. This is an intrinsic difference due to the different target, primers and probes between the two assay systems compared and the HPV types in the HPV array. It, however, did not reduce the clinical sensitivity of the assay as compared to Cobas 4800. It was observed that although the HPV 18 and/or other HR types were missed, other HPV types present in the infection were detected. Unfortunately, histology information was not available for all of the HPV DNA Array missed cases, however, HPV DNA Array did detect the high risk lesions, therefore, the analytical differences had little clinical bearing. One could suspect that the HPV types missed were not drivers of the infection, but little information is available in the literature.

The tendency of HPV DNA Array to have a lower agreement for HPV detection, but a very good agreement for detection of cervical intraepithelial neoplasia to the reference assays, could be explained by the higher number of viral copies in such lesions. Cervical intraepithelial neoplasia tends to have larger viral amounts and is, therefore, easier to detect [23]. It is then important that HPV assays which are meant to be used as screening tests have the right balance of clinical sensitivity and specificity [23].

We believe that the performance differences between HPV DNA Array and Cobas could occur due to differences in assay design (manual vs. automated DNA isolation), genotype spectrum detected, the target HPV gene (HPV DNA Array-E1, Cobas HPV test-L1 gene), and genotyping ability (full vs. partial genotyping). Evidently, when comparing HPV assays, an ideal high agreement is difficult to reach, as shown by Rebolj et al. [24]. In their paper on disagreement between HPV screening tests, they reported a 41% concordance for HPV positivity, irrespective of type, among 4 different fully validated HPV assays (Hybrid Capture 2, Cobas, CLART and APTIMA). The agreement among assays was even lower in the 30–65-year-old screening population, 29%. When focusing specifically on the Cobas test, which we also used in our study, it was observed that the agreement for HPV positivity, irrespective of type, to HC2, CLART, and APTIMA, varied between 50 and 70%. Although, Rebolj et al. did not analyze genotype-specific agreement, as in this study, and did not include HPV DNA Array in their comparison, the agreement of HPV DNA Array with Cobas of > 80%, underlines the good performance of HPV DNA Array for HPV detection.

Samples used in this study were received from HERMES study panel for validation purposes and do not represent a screening population. This explains the higher number of HPV positive and lesion positive samples than expected, approx. 80% within this study vs. approx. 10% within screening studies [20]. Within the HERMES study, 12.7% of samples were HPV positive [15], which is in concordance with other publications [20]. Furthermore, the HPV−/disease- population was underrepresented, leading to a lower specificity, due to a high number of HPV positive, but histologically normal samples. Therefore, the validation criteria set by Meijer et al. [23] could not be fulfilled, due to lack of samples from women undergoing regular cervical cancer screening. However, despite the sample background, the sensitivity of HPV DNA Array for detection of CIN2+ in women 30 years of age and older was > 90% of the reference assay, as required by Meijer et al. [23]. Further studies that will investigate if similar results can be reproduced in a screening population are justified.

The main limitation of this study was re-testing of the discordant samples with HPV DNA Array and the change of HPV results in 8 out of 90 re-tested samples. This change could occur during PCR and/or hybridization, as a pipetting error, reading error or contamination. Such re-testing was included in the analysis for validation purposes, but would not be feasible during routine diagnostics.

## Conclusion

This validation study has demonstrated the high potential of HPV DNA Array for detection of CIN2+/3+ lesions. HPV DNA Array has shown a very high clinical sensitivity of 100% for CIN2+ and 100% for CIN3+ detection, same as Cobas 4800; despite the analytical difference in detection of HPV 16 and 18 between the assays. Future studies are warranted to complete the validation and investigate the performance of HPV DNA Array in population-based screening and potentially in low resource settings.

## Data Availability

Not applicable. All relevant data are within the paper.

## Abbreviations

ASCUS: Atypical squamous cells of undetermined significance
CI: Confidence interval
CIN: Cervical intraepithelial neoplasia
FDA: Food and Drug Authority
HGSIL: High-grade squamous intraepithelial lesion
HPV: Human papillomavirus
HR: High risk
LGSIL: Low-grade squamous intraepithelial lesion
LR: Low risk
NPV: Negative predictive value
PCR: Polymerase chain reaction
PPV: Positive predicative value
